# Variations in Aging, Gender, Menopause, and Obesity and Their Effects on Hypertension in Taiwan

**DOI:** 10.1155/2014/515297

**Published:** 2014-11-10

**Authors:** Shu C. Chen, Tsai C. Lo, Jui H. Chang, Hsien W. Kuo

**Affiliations:** ^1^Public Health Bureau of Miaoli County Government, No. 6, Guofu Road, Miaoli City, Miaoli County 360, Taiwan; ^2^Department of Healthcare Administration, College of Medical and Health Science, Asia University, No. 500, Lioufeng Road, Wufeng, Taichung 413, Taiwan; ^3^Institute of Environmental and Occupational Health Sciences, National Yang-Ming University, No. 155, Section 2, Li-Nong Street, Taipei 112, Taiwan; ^4^School of Public Health, National Defense Medical Center, No. 161, Section 6, Minquan E. Road, Neihu District, Taipei 114, Taiwan

## Abstract

*Aim*. We assessed obesity, sex, menopause, and gender differences on hypertension in a Hakka-majority Taiwanese sample. *Methods*. 9621 subjects aged 20 and over participated in this community-based study. Trained nurses collected blood pressure (BP) measurements and anthropometric indices, including weight, height, hip circumference (HC), waist circumference (WC), body mass index (BMI), waist to height ratio (WHtR), and waist to hip ratio (WHR). *Results*. Levels of systolic and diastolic BP significantly increased at a dose-dependent relationship based on four anthropometric indices (BMI, WC, WHR, and WHtR); the slopes for SBP and DBP differed. After controlling for other covariates using multivariate logistic regression, we found the adjusted odds ratios (OR) of hypertension to be significantly related to the four anthropometric indices. Notably, the effect of obesity on the ORs for hypertension was considerably higher in premenopausal women, but we found no such phenomenon among men. BMI, WC, WHR, and WHtR had significant linear associations with BP. *Conclusion*. Obesity indices are significantly correlated with the risk of hypertension across gender and age, with BMI having the highest relative potency. The effect of obesity on the risk of hypertension is especially high in premenopausal women, implying a relationship between hormones and hypertension.

## 1. Introduction

Elevated blood pressure (BP) is associated with a higher risk of heart attack, heart failure, stroke, and kidney disease [1], and it is one of the components of metabolic syndrome [2]. Hypertension is a risk factor for many forms of cardiovascular disease (CVD) and other causes of death related to CVD [3, 4]. Thus, hypertention should be a concern for any modern nation. The significant increase of unhealthy lifestyle choices in Taiwan prompts the question: How do certain related indicators affect the risk of hypertension in the Taiwanese? Excessive dietary sodium intake, smoking, alcohol consumption, lack of physical activity, obesity, and hypertension have escalated in the Taiwanese population. A nationwide survey in Taiwan [5] showed the prevalence of hypertension, defined by a systolic BP > 140 mmHg or diastolic BP >90 mmHg, was 25% in men and 18% in women. This percentage was 47% in individuals of age ≥ 60 years, suggesting that the prevalence of hypertension in Taiwan increases with age.

For this trend, the age-related rise in systolic BP from ages 40 to 80 years was steeper in women than in men. Systolic BP increases after the age of 60, whereas diastolic BP decreases, a phenomenon causing an increase in pulse pressure. The rise in pulse pressure is the result of a decrease in the extensibility of the aortic wall and is associated with the progression of atherosclerosis [5]. We referenced the prevalence rates of hypertension in 10 developed countries and found it to be higher in men than in women under the age of 60. After 60, this prevalence rate is higher in women [6]. Reckelholff (2001) reviewed human gender differences in the regulation of BP and found men to be at a greater risk for CVD than age-matched premenopausal women [7]. Although the mechanisms responsible for the gender differences in BP control are unclear, we have significant evidence that hormones, such as testosterone or estrogen, play an important role in gender-associated differences in blood pressure regulation [7–9]. The level of estrogen rises and falls unevenly during perimenopause.

This hormonal effect on blood pressure regulation is a remarkable topic in regard to the risk of hypertension. Menopause often occurs in a woman's 40s in Taiwan, whereas the average age of menopause is 51.4 in the United States [10]. Menopause is accompanied by a dramatic rise in the prevalence of hypertension in women, suggesting both scientific phenomenon—a protective role of endogenous estradiol in BP—and a social phenomenon—a particularly severe and early problem of hypertension for Taiwanese women [8, 11]. The loss of estrogen that accompanies menopause contributes to the development of obesity and high BP. Despite the implications of early menopause, few studies have stratified menopause when researching the effects of obesity on hypertension. In this community-based study focusing on the Taiwanese, a group of people who differ from the citizens of most modern nations in menopause and hypertention, we assessed the age and gender effects on hypertension, analyzing the differences of these effects on premenopausal and postmenopausal women.

## 2. Materials and Methods

### 2.1. Study Population

The Institutional Review Board (IRB) of Yang-Ming University approved our cross-sectional study. Using probability proportional to population size (PPS) methods, we randomly selected, from the total population of Miaoli County, 9261 participants (response rate of 92.6%) of ages 20 and above. We notified all participants that they had the right to reject or withdraw from this study at any time and that all information acquired in this study that could identify them would remain confidential. The questionnaire included demographic characteristics (gender, age, education, occupation, and ethnic group) and was administered by trained nurses in health centers.

### 2.2. Anthropometric Measurement

During the questionnaire interview, each participant provided measurements of height, weight, waist circumference (WC), and hip circumference (HC), all without shoes and with minimal clothing. We calculated BMI in kg/m^2^; WHR by WC/HC in centimeters; and WHtR by WC/height in centimeters. Weight and height were obtained using a stadiometer and digital or balance-beam scale. WC was measured at the midline between the lowest costal margin and the superior posterior iliac crest in a horizontal plane. HC was measured at the level of the greater trochanter. Height and WC were recorded to the nearest 0.1 cm and body weight to the nearest 0.1 kg. The Taiwan Bureau of Standards, Metrology and Inspection verified all tools of measurement. We calculated obesity and defined its cutoffs according to the standards set by the Department of Health in Taiwan. Cutoffs for high BMI were 25 for females and 27 for males. Cutoffs for high WHR were 0.9 for males and 0.8 for females. Cutoffs for high WHtR were 0.45 for females and 0.48 for males. Cutoffs for high WC were 85 cm for females and 90 cm for males [12].

### 2.3. Blood Pressure Measurement

Using the standardized mercury sphygmomanometer with manually inflated cuff of suitable size and a stethoscope, nurses—supervised by medical doctors—measured BP via the participant's right arm after the participant had rested for 20 minutes in a sitting position. SBP was determined by the onset of the “tapping” Korotkoff sounds (K1) and the fifth Korotkoff sound (K5); DBP was determined by the disappearance of Korotkoff sounds. Hypertension is generally defined as a systolic BP of >140 mmHg, a diastolic BP of >90 mmHg or use of antihypertensive medications [13, 14].

### 2.4. Statistical Analyses

We performed statistical analyses using SPSS, version 20. We compared differences in age, gender, and different body composition measures (weight, height, BMI, WC, HC, WHR, and WHtR) between the healthy and hypertensive populations. Stratified by sex, obesity levels were classified by five categories on five anthropometric indices (BMI, WC, HC, WHR, and WHtR), which were plotted with the increase levels of systolic BP and diastolic BP—an analysis performed via multivariate linear regression adjusted for covariates. We defined the postmenopausal stage with the cutoff point of age 50. Using multivariate logistic regression analysis adjusted for educational level and race, we computed the independent impact of menopause and gender on the odds ratios (ORs) of hypertension with 95% confidence intervals (CIs). We took *P* values of under 0.05 to denote statistical significance.

## 3. Results

Table 1 shows demographic information of both the healthy and hypertension population. Significant differences in the demographic and obesity indices in two groups are palpable. A high percentage of women and the elderly fell in the hypertension group. As we expected, obesity indices, including BMI, WC, HC, WHR, and WHtR, were significantly high in the HP group.

Table 2 shows the results of multiple linear regression adjusted for age, race, education, and occupation. The anthropometric indices were dose-dependently correlated with systolic BP and diastolic BP for each gender. When we classified BMI and WC by five levels, we found the slopes of systolic BP and diastolic BP with BMI and WC levels to increase in men. Obese persons (defined as BMI over 30) had high levels of systolic BP and diastolic BP, with 16.69 and 12.53 mmHg in men and 12.30 and 10.18 mmHg in women. The differences in BP between the obese group and those of groups with BMI < 18 were significant. Similarly, obese persons with HC of 90 cm or over had high systolic BP and diastolic BP: 10.05 and 7.09 mmHg in men and 9.85 and 6.59 mmHg in women, again significantly higher levels than those in the HC < 72 cm group. WHR and WHtR were also significantly correlated with systolic BP and diastolic BP by gender; however, we found no difference in the increase of the slope on the levels of systolic BP and diastolic BP in men and women. Overall, regardless of which obesity indicator is used, the trends for SBP and DBP versus obesity remained the same.

Table 3 shows our multivariate logistic regression adjusted for education levels and race. We found that obesity, as determined by four anthropometric indices, was significantly correlated with the risk of hypertension across gender and age. Overall, regardless of which obesity indicator is used, the trends for SBP and DBP versus obesity remained the same. However, the relative potency of those trends varied by which indicator we employed. BMI showed the highest potency. In addition, the potency for SBP's trend was higher than that of DBP, regardless of sex. We classified age by seven strata. As per our multivariate logistic regression adjusted for BMI, education levels, and race, the risk of hypertension was dose dependent with the age stratum for each gender (Table 3). The risk of HP in each age stratum for women was 1.56 to 2.57 times higher than that of men. For women, the risk for hypertension increases with age, with the notable cutoff point of menopause—age 50—marking a transition in this value. When comparing sexes after the age of 50, we found that women had a lower risk of hypertension than did men. This trend is likely explained by the hormonal changes that come with menopause. This was true for all four indicators of obesity. For this data, we created an obesity score, which we defined as the sum of abnormal obesity indicators—a number that ranged from 0 to 4. We found that the risk of hypertension was dose dependent with this obesity score, a fact that held true for all sex and age groups.

**Figure 1:**
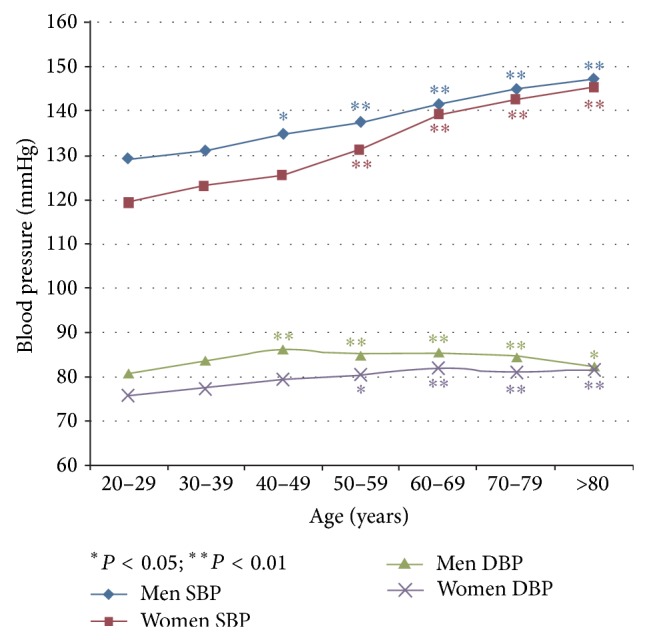
Age affected systolic BP and diastolic BP adjusted for BMI, education levels, and race by gender using multivariate linear regression (group with ages 20–29 used as baseline data which is compared with other age strata groups).

As per our multivariate linear regression adjusted for BMI, education levels, and race, we found the systolic BP levels to be dose dependent with the age stratum for each gender (Figure 1). However, the diastolic BP levels among men considerably increased from ages 20 to 50 and then decreased after age 50. Diastolic BP levels among women increased from ages 20 to 70 and then decreased after age 70. This is in contrast with the slope SBP, which increases after 50 for women. Evidently, systolic BP and diastolic BP levels are consistently higher in men than in women at similar ages, adjusted for covariates. Remarkably, the increase in SBP is age dependent, whereas that for DBP is not. Figure 1 also gives a hint to the interactions between age and sex, yet we did not statistically test these interactions.

## 4. Discussion

Despite the knowledge of the association between increased rates of CVD and hypertension as well as metabolic syndrome in Taiwan, few studies have specifically assessed the obesity of the effect on BP and hypertension by gender. Our study found the risk (ORs) of developing hypertension to be significantly related to the four anthropometric indices (BMI, WC, WHR, and WHtR) by gender. Overall, regardless of which obesity indicator is used, in the increase of the slopes on the levels of systolic BP and diastolic BP in men and women remain similar trends. However, the relative potency of those trends varied by which indicator we employed. BMI showed the highest potency, indicating that it is significantly correlated with adiposity and can predict the body fat percentage adequately when age and gender are considered.

Most notably, the effect of obesity on the ORs for hypertension was considerably higher in premenopausal women (aged 50 and lower), but no such phenomenon was found among men. Although the mechanisms responsible for the gender differences in BP control are unclear, we have significant evidence that androgens, such as testosterone, play important roles in the gender-associated differences in BP regulation [7]. Previous studies using the technique of 24-hour ambulatory BP monitoring have shown that BP is higher in men than in women at similar ages by approximately 6 to 10 mmHg, until the ages of 70 to 79 years, when BP was similar for men and women [15, 16]. During childhood, BP increases in both boys and girls along with the increase in age, a phenomenon discovered using ambulatory BP monitoring techniques. However, after the onset of puberty, boys have higher BP than do age-matched girls. At ages 13 to 15 years, systolic BP was approximately 4 mmHg higher in boys than girls, and at ages 16 to 18 years, boys had higher systolic BP than girls by 10 to 14 mmHg [17].

Unfortunately, the above-mentioned studies did not further adjust for the covariates of BP. In our study, we showed systolic BP levels to be dose dependent with age strata, after adjusting for BMI, educational level, and race via multivariate linear analyses for each gender. However, diastolic BP levels among men quickly increased from ages 20 to 50 and then decreased after age 50. We found the same increase-decrease pattern to be true for women, except that the cutoff age was 70 instead of 50. Evidently, systolic BP and diastolic BP levels are consistently higher in men than in women at similar ages, after adjusting for covariates. The difference in systolic BP and diastolic BP levels in both men and women showed high variations, from 1 to 10 mmHg among different age strata. Adjusting for covariates of BP using multivariate linear regression, we found that systolic BP and diastolic BP among obese men people (BMI > 30) were 2.4 mmHg and 4.4 mmHg higher than those for women.

After menopause, however, BP increases in women to levels even higher than those in men. We found that blood pressure increased with age in both men and women, which increased the risk of HP 1.56- to 2.57-fold. For women, BP generally increases after menopause, which we explain as a result of hormonal changes. Others think the increase in obesity in menopausal women might play a greater role than the hormonal changes [7, 18]. Menopause-related hormonal changes can indeed lead to both weight gain and an increase in how blood pressure reacts to salt in a person's diet, which, in turn, can lead to higher BP. In addition, some types of hormone therapy (HT) for menopause also might contribute to increases in BP. As estrogen decreases, the walls of a woman's blood vessels can become less flexible, causing BP to rise, which increases the risk factors for stroke and heart disease. With the decline in estrogen levels, the risk factors for heart disease, especially high BP, become more apparent [19, 20].

The 2007 Taiwanese national survey showed the overall prevalence rate of high BP to be 27.7% (30.3% for men and 25.4% for women). That rate increased to 47% among individuals of age ≥ 60 years [7]. In this study, we show an even higher prevalence of hypertension: 34.7% in Miaoli County (39.2% for men and 31.0% for women). In the study, over 60% of the participants were Hakka, a subgroup of Han Chinese. Prevalence of hypertension among Hakka with type 2 diabetes mellitus is also more prevalent than in their Fukienese counterparts [21]. Hakka have a significantly higher prevalence of hypertension (56.8% versus 52.9%; *P* < 0.001) with an adjusted odds ratio of 1.19 (1.11–1.27) [22]. The underlying etiology leading to the ethnic differences in the prevalence of hypertension is still not known. The dietary patterns in the Hakka could play some role because they consume more preserved vegetables and use large amounts of lard in their cooking, suggesting an increased salt intake and propensity to the development of atherosclerosis [23]. Because the Hakka people are culturally predisposed to consuming high-energy food to supply them with the strength needed in the work day, their food is oily and salty.

In Eastern Taiwan, 27.4% of 833 adults over 30 with disabilities were diagnosed with hypertension. After we controlled for marital status and type and level of disability, we found the predictive factors of age (OR = 2.45), with being overweight or obese (as per BMI) (OR = 6.72), WC (OR = 1.64), and vegetable/fruit intake (OR = 0.61) to be significantly correlated with the risk of hypertension [24]. Similarity, our study shows that the risk of hypertension significantly increased with a dose-dependent relationship with four anthropometric indices. However, Lin's study did not elaborate on these relationships between risk of hypertension and obesity or on the differences due to gender and age. In our study, we found age groups to be dose-dependently correlated with the risk of hypertension across gender, with the incremental risk of hypertension in women being higher than that of men at each age group. Gender differences on the risk of developing hypertension varied by age.

We explain our findings with how uncontrolled hypertension (HTN) varies by gender. Specifically, women have been shown to have worse rates of BP control. Previous studies have suggested that women have lower rates of hypertension control and that these differences can vary with age. Yet studies have shown that men tend to have lower rates of hypertension control compared to women (41.2% versus 45.7%, adjusted OR = 0.93, and 96% CI 0.91–0.95) [25, 26].

## 5. Strengths and Limitations

Our findings indicate not only that SBP and DBP levels are significantly correlated with anthropometric indices but also that gender differences in the risk of developing hypertension vary by age. However, the use of a cross-sectional design was a limitation of this study, restricting our ability to establish causal relationships. Although we based our sampling protocol on the PPS technique, other factors might contribute to the development of hypertension, including family history of hypertension, duration of diabetes, dietary components, socioeconomic status, physical activity, and genetic variations, all factors not completely collected. Nevertheless, previous studies have discovered lifestyle habits detrimental to BP and hypertension, including the consumption of alcohol, betel nut chewing, and high-fat diets. Secondly, much evidence shows that BP levels in women generally increase after menopause. To accurately define the timing of menopause, which is defined as a woman's last menstrual period, is difficult. Menopause usually occurs around age 51 but can range from 45 to 55 [10]. In Taiwan, women face the same fears and discomforts about menopause as women in Western nations, but Taiwanese women are often reluctant to talk to their interviewers about the timing of menopause. We defined the postmenopausal stage with the cutoff point of age 50, which could not have defined menopause for all the women in the sample.

Future studies should investigate two aspects. First, researchers should investigate the interaction effects of the indicators we found to be correlated with hypertension, namely, age, sex, menopause, and obesity. Second, researchers in Taiwan should stratify for ethnic group (e.g., Hakka versus Hoklo) to discover differences in both hypertension and the indicators correlated with hypertension, as well as any other lifestyle differences that would prove useful in the prevention of hypertension.

## 6. Conclusions

Obesity indices, including BMI, WC, HC, WHR, and WHtR, are significantly high in the HP group. The risk of hypertension is dose dependent with age strata for each gender, and the risk of HP in each age stratum in women is 1.56- to 2.57-fold higher than that of men. Women over 50 have a risk of HP 5.34 to 13.82 times higher than men and a risk of HP 2.52 to 5.78 times higher than women at age < 29. Systolic BP levels are dose dependent with age strata for each gender. Similarity, diastolic BP levels among women aged 20 to 70 increase and then decrease after 70. Obesity, regardless of which of the four anthropometric indices included defines it, is significantly correlated with the risk of hypertension for each gender and age stratum. The obesity score is also dose-dependently correlated with the risk of HP for each gender. The effect of obesity on the risk of hypertension is especially high in premenopausal women.

## Figures and Tables

**Table 1:** A comparison of demographic information between healthy and hypertension groups.

	Healthy group (*N* = 6282)	Hypertension group (*N* = 3339)	*P*
Gender (%)			<0.001
Men	57.7%	48.8%	
Women	42.3%	51.2%	
Age > 50 year (%)	55.8%	80.8%	<0.001
Mean (SD)	49.9 (15.3)	60.2 (13.9)	<0.001
BMI	24.0%	36.5%	<0.001
Mean (SD)	23.5 (3.4)	251 (3.6)	<0.001
WC (%)	22.7%	40.6%	<0.001
Mean (SD)	79.5 (10.6)	85.0 (10.6)	<0.001
HC mean (SD)	93.7 (9.7)	95.6 (9.6)	<0.001
WHR (%)	51.6%	70.1%	<0.001
Mean (SD)	0.85 (0.13)	0.89 (0.11)	<0.001
WHtR (%)	69.2%	86.5%	<0.001
Mean (SD)	0.49 (0.07)	0.53 (0.07)	<0.001

BMI: >27 kg/cm^2^ for male; >25 kg/cm^2^ for female; WC (waist circumference): male: >90 cm for female: >85 cm; WHR: male: >0.9 for female: >0.8; and WHtR: male: >0.48 for female: >0.45.

HC: hip circumference, WHR by WC/HC in centimeters; WHtR by WC/height in centimeters.

**Table 2:** Anthropometric indices dose-dependently correlated with systolic BP and diastolic BP across gender, adjusted for age, race, education, and occupation using multiple linear regression.

	Men (*N* = 4364)		Women (*N* = 5257)	
	SBP	DBP	SBP	DBP
BMI (kg/cm^2^)				
<18	Reference	Reference	Reference	Reference
18.01–24	2.65	3.64^*^	0.64	0.31
24.01–27	6.38^**^	5.01^**^	2.18	3.07^*^
27.01–30	10.66^**^	8.42^**^	5.47^**^	5.37^**^
>30	16.69^**^	12.53^**^	12.30^**^	10.18^**^
Waist circumference (cm)				
<71.99	Reference	Reference	Reference	Reference
72–77.99	3.63^**^	2.02^**^	1.93^*^	1.56^**^
78–83.99	5.66^**^	3.93^**^	3.25^**^	2.67^**^
84–89.99	8.22^**^	5.50^**^	5.47^**^	4.11^**^
>90	10.05^**^	7.09^**^	9.85^**^	6.59^**^
Hip circumference (cm)				
<87.99	Reference	Reference	Reference	Reference
88–91.99	3.02^**^	1.93^**^	0.30	0.84
92–95.99	5.06^**^	2.96^**^	2.31^**^	1.83^**^
96–99.99	6.55^**^	3.72^**^	4.82^**^	3.38^**^
>100	8.34^**^	5.53^**^	6.34^**^	4.37^**^
WHR				
<0.79	Reference	Reference	Reference	Reference
0.80–0.84	0.60	−0.33	0.46	−0.17
0.85–0.89	1.57^**^	0.90	0.13	0.18
0.90–0.94	2.93^**^	2.71^**^	0.38	−0.17
>0.95	2.05^**^	2.13^**^	2.98^**^	1.30^*^
WHtR				
<0.45	Reference	Reference	Reference	Reference
0.45–0.49	1.21	0.04	1.33^*^	0.10
0.49–0.52	2.53^**^	1.29^**^	1.96^**^	0.70
0.52–0.56	3.33^**^	2.02^**^	3.62^**^	0.35
>0.56	4.07^**^	2.60^**^	6.25^**^	1.92^**^

^*^ *P* < 0.05; ^**^ *P* < 0.01.

**Table 3:** Obesity using anthropometric indices correlated to the risk (odds ratio) of hypertension, adjusted for education levels and race across gender and age groups, using multivariate logistic regression.

	Men (*N* = 4364)		Women (*N* = 5257)	
	Aged < 50 (*N* = 1507)	Aged > 50 (*N* = 2857)	Aged < 50 (*N* = 1911)	Aged > 50 (*N* = 3346)
BMI	2.39^**^ (1.84–3.10)	2.23^**^ (1.84–2.70)	2.53^**^ (1.89–3.40)	1.88^**^ (1.62–2.18)
WC	2.08^**^ (1.62–2.67)	1.91^**^ (1.64–2.24)	1.93^**^ (1.36–2.73)	1.55^**^ (1.33–1.80)
WHR	1.71^**^ (1.35–2.17)	1.36^**^ (1.16–1.59)	2.01^**^ (1.50–2.68)	1.81^**^ (1.52–2.15)
WHtR	2.08^**^ (1.61–2.67)	1.80^**^ (1.49–2.18)	2.86^**^ (2.02–4.04)	1.73^**^ (1.35–2.23)
Obesity scores				
0	1	1	1	1
1	1.31 (0.94–1.84)	1.57^*^ (1.22–2.04)	2.02^*^ (1.26–3.23)	0.82 (0.58–1.15)
2	1.79^*^ (1.25–2.56)	1.42^*^ (1.11–1.81)	2.50^**^ (1.63–3.83)	1.67^**^ (1.23–2.27)
3	2.19^**^ (1.49–3.20)	2.18^**^ (1.68–2.81)	3.86^**^ (2.36–6.31)	2.03^**^ (1.47–2.81)
4	3.56^**^ (2.46–5.12)	3.69^**^ (2.77–4.92)	4.94^**^ (3.03–8.06)	2.57^**^ (1.86–3.53)

BMI: >27 kg/cm^2^ for men; >25 kg/cm^2^ for women; WC: >90 cm for men >85 cm for women; WHR: >0.9 for men; >0.8 for women; and WHtR: >0.48 for men; >0.45 for women; obesity scores are calculated as the total number of abnormal items from the four indicators of obesity.

^*^ *P* < 0.05; ^**^ *P* < 0.01.
